# TFEBexplorer: An integrated tool to study genes regulated by the stress-responsive Transcription Factor EB

**DOI:** 10.1080/27694127.2022.2097822

**Published:** 2022-07-21

**Authors:** Rossella De Cegli, Diego Carrella, Diletta Siciliano, Gennaro Gambardella, Gennaro Napolitano, Chiara Di Malta, Andrea Ballabio, Diego di Bernardo

**Affiliations:** a Telethon Institute of Genetics and Medicine (TIGEM), via Campi Flegrei, 34, 80078 Pozzuoli (Naples), Italy; b Department of Chemical, Materials and Industrial Engineering, University of Naples ‘Federico II’, 80125 Naples, Italy; c Medical Genetics Unit, Department of Medical and Translational Science, Federico II University, Via Pansini 5, 80131 Naples, Italy; d Jan and Dan Duncan Neurological Research Institute, Houston, TX 77030, USA; e Department of Molecular and Human Genetics, Baylor College of Medicine, Houston, Texas 77030, USA

**Keywords:** autophagy, binding site, gene expression, lysosome, TFEB

## Abstract

TFEBexplorer is a comprehensive web-tool to study genes modulated by the transcription factor EB (TFEB), a master regulator of lysosomal biogenesis and autophagy, that integrates multiple datasets following genetic perturbation of TFEB with experimentally validated and bioinformatically-predicted binding sites across multiple species. Given one gene, or a set of genes of interest, in input (query), the results are displayed in different panels, each reporting results of experiments or bioinformatic analyses.

TFEBdb is a unique resource for those interested in understanding the effect of TFEB on a set of genes of interest. The database offers an intuitive interface and results are displayed in an easy-to-interpret format offering information on the expression changes following inducible over-expression, knock-down and knock-out of TFEB across cell lines and species together with experimentally validated and bioinformatically predicted binding sites in the proximal promoter of the gene(s) of interest. The TFEBdb homepage can be found at http://tfeb.tigem.it.

**Abbreviations** ChIP-seq: chromatin-immunoprecipitation followed by sequencing; CLEAR: Coordinated Lysosomal Expression and Regulation; DMEM: Dulbecco’s modified Eagle’s medium; FDR: False discovery rate; GEPs: gene expression profiles; PBS: phosphate-buffered saline; RMA: robust multiarray average TFEB: The transcription factor EB; TFEBdb: database; ToA: time of activation of the gene.

## Introduction

The transcription factor EB (TFEB) is a basic Helix-Loop-Helix (bHLH) leucine zipper transcription factor, member of the microphthalmia/ transcription factor E (MiT/TFE) family of transcription factors, which also includes MITF, TFE3, and TFEC proteins [1]. As in other bHLH-Zip transcription factors, the MiT/TFE members share a basic DNA-binding domain and an HLH plus a leucine zipper domain important for dimerization [2]. The MiT/TFE members form homo- and hetero-dimers to activate transcription [3]. TFEB and TFE3 play a crucial role in the regulation of lysosomal and autophagy-related genes [4–6], while MITF regulates melanosomal biogenesis [7]. All three genes play an important role in the regulation of cell metabolism [8]. TFEB activity is largely controlled by its subcellular localization, which is mainly regulated by mechanistic target of rapamycin complex 1 (mTORC1)-dependent phosphorylation; in presence of nutrients, TFEB is mainly cytoplasmic but, upon stresses such as nutrient depletion, inactivation of the mTORC1 complex causes its rapid translocation to the nucleus [9–11] where it activates a tissue-specific transcriptional program. The binding to DNA of the MiT/TFE members is mediated by the recognition of the E-box sequence (CACGTG) [1], which is typically recognized by other bHLH-Zip transcription factors. However, MiT/TFE factors preferentially recognize a particular type of E-box that is flanked by specific nucleotide residues, known as the “Coordinated Lysosomal Expression and Regulation” (CLEAR) motif (GTCACGTGAC) [12-14].

The TFEB-regulated transcriptional program coordinates the biogenesis of autophagosomes and lysosomes inducing a striking increase in autophagy flux via regulation of the expression of genes involved in different steps of the autophagy process, such as genes important for: autophagy initiation (BECN1, WIPI1, ATG9B, and NRBF2); autophagosome membrane elongation (GABARAP, MAP1LC3B, and ATG5); substrate capture (SQSTM1) and autophagosomes trafficking and fusion with lysosomes (UVRAG, RAB7) [5]; and lysosomal biogenesis and degradation [4, 5, 14]. Two family members, TFE3 and MITF, were also identified as regulators of autophagy and lysosomal biogenesis [15, 16]. In addition to its role in lysosomal biogenesis and autophagy, TFEB is involved in the regulation mTORC1 signaling [17], in the integrated stress response (ISR) [13, 18] and in the peripheral clock machinery [19]. Despite many target genes are similarly modulated by TFEB in different cell types, TFEB has been shown to have specialized functions in different tissues: in the liver, in addition to lysosomal function and autophagy, TFEB regulates genes important for the control of lipid metabolism, including lipophagy and fatty acid oxidation [20] [21]; in osteoclasts, TFEB deletion specifically results in impaired function and increased bone mass, suggesting that TFEB regulates bone resorption [22]; in skeletal muscle, TFEB directly controls glucose homeostasis and mitochondrial biogenesis independently from its role in the modulation of autophagy [23]; in macrophages, TFEB and TFE3 control the expression and secretion of pro-inflammatory cytokines, thus modulating inflammatory and immune responses ([24, 25]).

Owing to its role as a master modulator of cell catabolism, transient induction of TFEB-mediated transcriptional programs may have beneficial effects in several disease conditions characterized by impaired lysosomal activity or autophagy [26]. Several compounds have been suggested as a potential TFEB activator: polyphenols such as curcumin are able to activate the TFEB-lysosome pathway for induction of autophagy [34]; “resveratrol” attenuates endothelial oxidative injury by inducing autophagy via the activation of TFEB [35]; trehalose has been recently demonstrated to induce TFEB nuclear translocation and autophagy in models of motoneuron degeneration [36], and it was found also able to reduce atherosclerotic plaque burden [37].

On the other hand, constitutive TFEB overexpression or activation has been linked to cystic disorders and cancer. Indeed, up-regulation or constitutive activation of MiT/TFE genes supports the energy-demanding growth and metabolism of different types of cancer, including renal cell carcinoma (RCC), Birt–Hogg–Dubé syndrome, pancreatic ductal adenocarcinoma, and melanoma [25] [29-33]. The Open Targets Platform [27, 28], includes more than 300 diseases and/or phenotypes associated to TFEB, such as cystic disorders and cancer.

**Figure 1.**
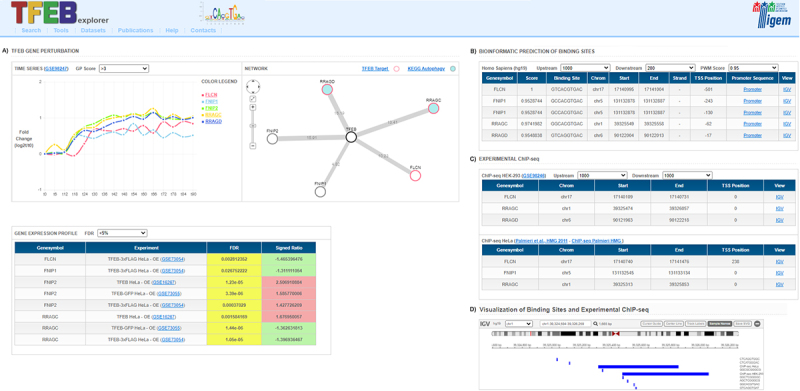
**The TFEBexplorer interface**. Given a set of genes in input (query), the results of the Search are displayed in different panels, each reporting results of experiments or bioinformatic analyses: **(A)** TFEB gene perturbation panel includes: a time-series box with expression changes of the query genes over time following inducible TFEB overexpression in human HEK293 cells; a network box with a network representation showing query genes as nodes (red=direct TFEB targets; cyan filled circles=autophagy pathway gene); and a gene expression profile box listing the significant differentially expressed genes in the query in the available human and mouse transcriptional datasets; **(B)** Bioinformatic prediction panel reports the genome-wide predictions of TFEB CLEAR binding sites; **(C)** Experimental ChIP-seq panel lists the TFEB binding sites in HEK293 and HeLa cells identified in published Chromatin immunoprecipitation followed by sequencing experiments (ChIP-seq); **(D)** Visualization of binding site panel is opened by clicking on the “IGV” link and shows a gene-wise graphical representation of the binding sites in panel B and C along the genomic region in the Integrative Genome Browser.

Despite the intricate network of genes regulated by TFEB and the pleiotropic roles this transcription factor plays in numerous tissues, as of today, no systematic collection of genes modulated and targeted by TFEB exists. Here, we present the first online tool, available at http://tfeb.tigem.it, and schematically depicted in Figure 1.

## Results and Discussion

TFEBexplorer is built on an integrated collection of experimental data and bioinformatics predictions to yield the first comprehensive resource for the study of genes regulated by TFEB. The databases includes the following publicly available dataset: (i) 18 gene expression profiles (GEPs) in human (GSE16267, GSE73055, GSE73054) and 36 GEPs in mouse (GSE41139, GSE41140, GSE62975, GSE62976, GSE62977, GSE63376) following TFEB overexpression, down-regulation or knock-down; (ii) two TFEB chromatin-immunoprecipitation followed by sequencing (ChIP-seq) in two human cell lines (HeLa [14] and Hek293[13]) and (iii) a genome-wide bioinformatic prediction of TFEB binding sites across three species (human, mouse and rat). A brief description and relevant references for each dataset is reported in Supplementary Table 1 and in the online website (“Datasets” section).

In brief, human specific datasets include time-resolved transcriptome profiles (GSE98247) of a stable clone with inducible overexpression of TFEB in Hek293 cells [13]; transcriptional profiles in HeLa cells following wild-type TFEB transient transfection (GSE16267) and stable overexpression of the fusion construct TFEB-GFP ([38], GSE73055). We also added a previously unpublished dataset of transcriptional profiles in HeLa cells with stable expression of the fusion construct TFEB-3xFLAG from the CMV promote measured by Affymetrix Gene-Chip hybridization experiments (GSE73054).

Mouse specific datasets include transcriptional profiles from three tissues: (*liver*) wild-type mice or mice knocked-out for PPARα (peroxisome proliferator activated receptor alpha) were injected with an adenoviral vector that expresses human *TFEB* (HDAd-*TFEB*) under control of the liver-specific promoter PEPCK [21] (GSE41139 and GSE41140); (*skeletal muscle)* gene expression profiles include both TFEB gain- and loss-of-function approaches (SuperSeries-GSE62980): the overexpression of *Tcfeb*, (the murine homolog of the human *TFEB*), in adult mouse skeletal muscle was achieved by means of intramuscular viral-mediated gene transfer using the adeno-associated virus (AAV) system (GSE62975); for loss-of-function studies (GSE62976), a mouse model with muscle-specific conditional TFEB KO was generated by crossing *Tcfeb* floxed [21] with MLC1f-Cre transgenic mice [39]. Muscle fibers from TFEB KO animals and controls were then profiled by Affymetrix microarray experiments, as previously described [39]; (*kidney*), transcriptome analysis by Affymetrix microarrays was performed on total RNA of kidneys from mice overexpressing *Tcfeb* in the kidney (heterozygous KSP_CRE/KSP_Tcfeb mice) and from matched controls (KSP_CRE mice) at two different time-points of mice development (P0 and P14) (GSE62977, GSE63376) [40].

The TFEBexplorer web-tool is divided into six sections: “Search”, “Tools”, “Datasets”, “Help”, “Publications” and “Contacts”:

The “Search” page is the home page, and it allows the user to choose the species of interest and to enter a set of genes in the Query Box as official gene symbols or aliases (up to 10 genes are allowed), which autocompletes for ease of use. The gene set of interest in the search box is automatically updated if the user decides to change the species of interest. Examples of gene sets are also present in the home page; by clicking on the example gene set name, the gene list is automatically added to the Query Box . The results of the query are displayed across different panels as depicted in Figure 1:

- the “TFEB gene perturbation” panel reports the expression dynamics of the genes following inducible overexpression of TFEB in Hek293 cells (GSE98247, [13]). The time-course gene expression profile experiments were performed by RNA-seq on total RNA extracted at time 0, in presence of tetracycline to evaluate the basal expression level of TFEB, and at consecutive time point (from 6 to 90hrs) following the removal of tetracycline to evaluate the effect of TFEB overexpression. In this section, there is also a TFEB network diagram to graphically depict the genes that are significantly modulated in the time-series data; significance is measured by a Gaussian Process (GP) approach [41] and results can be filtered by GP score. In the network diagram, TFEB targets [13] are shown as red circles. By clicking on the circle it is possible to access the gene card website; an additional link is shown for those genes belonging to the KEGG autophagy [42] pathway, which are shown as filled cyan circles.
- the “Gene Expression Profile” panel shows the fold-change, as signed ratio value, for the query genes (negative values colored in green for inhibition, and positive values colored in red for induction) in all of the gene expression dataset included in TFEBexplorer, together with its significance reported as False Discovery Rate (FDR).
- the “Bioinformatic Prediction of binding sites” panel reports the predicted CLEAR binding motives in the proximal promoter of the query genes.
- the “Experimental ChIP-seq” panel lists the chromosomal coordinates of binding sites in the promoter as found in two different ChIP-seq datasets performed in HeLa [14] and Hek293 [13]. The panel is interactive with gene names hyperlinked to the GeneCard website [43], the dataset ID hyperlinked to the GEO expression omnibus database [44], while the promoter sequence and the bioinformatically predicted CLEAR binding sites can be visualized by clicking on the “IGV” hyperlink.

The “Tools” page is used to perform analyses on multiple genes and includes three type of analyses: by clicking on “Prediction of Binding Sites”, it is possible to paste a list of genes of interest (with no limit in length of the gene list) and obtain the bioinformatics prediction of the CLEAR binding sites in their promoters; the “Gene Expression Profile” tool allows to query a set of genes (no limit in number) to check the effect of TFEB perturbations on its expression across all the datasets included in the database; finally by clicking on the “Time Series TFEB Network” it is possible to visualise the genes modulated by TFEB overexpression (dataset GSE98247) as a network.

The “Dataset” page displays a version of Supplementary Table 1 with hyperlinks to download any of the datasets used in the database. The “Help” page displays a document with a step-by-step guide to the use of the database, while the “Contacts” page reports name and email of contact persons for queries related to the use of the database.

We introduced two demo sets as query examples for all the tools: “mTOR regulators” and “Autophagy-lysosomal pathway”. In details, in the “mTOR regulators” gene set, we included the previously established TFEB targets [17] RRAGC, RRAGD, FLCN, and two homologous FLCN-binding proteins FNIP1 and FNIP2, which are known modulators of mTORC1 [45]; the “autophagy-lysosomal” set includes two lysosomal hydrolases (GNS and NEU1), three v-ATPase subunits (ATP6V0D1, ATP6V0A1 and ATPV1H), the lysosomal membrane protein LAMP1, the vesicle mediated protein VPS18 [14], and the small GTPase RAB7 [14].

We believe TFEBexplorer will be a useful and user-friendly resource for researchers interested in the physiological and pathological roles of TFEB and the MiT/TFE members and in the transcriptional regulation of lysosomal biogenesis and autophagy.

We will update the database yearly by introducing relevant datasets as these become available. In a future version of TFEBexplorer, we will also add data for two additiona species *Caenorhabditis elegans* [21], and *Oryzias latipes* [46](i.e. Medaka fish).

## Materials and Methods

The website (http://tfeb.tigem.it/) is implemented in PHP and Javascript. The database is implemented in PostgreSQL. This resource is usable with any browser (Table 1) and has been optimized to be used with PC or Tablet.

**Table 1.** Browser compatibility

OS	Version	Chrome	Firefox	Microsoft Edge	Safari
Linux	Ubuntu 20.04	96.0.4664.45	94	n/a	n/a
MacOS	Mojave 10.14.6	96.0.4664.55	68.0.2	n/a	14.1.2
Windows	10	96.0.4664.45	94.0.1	96.0.1054.29	n/a

## Bioinformatic prediction of TFEB binding sites and visualization of results

To bioinformatically identify the CLEAR motifs in gene promoters, we downloaded genomic sequences including 2000 bp upstream and 500 bp downstream from the transcription start site (TSS) for all the known genes for human, mouse, and rat species. The sequences were downloaded from UCSC Genome Browser website [47, 48] using these genome versions: hg19 for human, mm10 for mouse and rn6 for rat. For the determination of the binding sites, we applied the package match-PWM by Biostrings with default parameters while the threshold for the PWM Score (min.score) was set to 0.85. [49]. The position-weight matrix of the TFEB consensus binding sequence was identified using the Weeder tool 2.0, as previously described [13] and it can be downloaded from the “DATASETS” section of the TFEBexplorer website. The algorithm match-PWM establishes an enrichment score (PWM Score) for each sequence referred to the TFEB matrix. The PWM score ranges from 0 to 1, with 1 corresponding to the maximum score that can be obtained with that PWM. For more details on the PWM method and a recent review we refer the reader to the relevant literature [50]. The visualization of the binding sites on the website is performed by making use of the Integrative Genomics Viewer (IGV), a high-performance, easy-to-use, interactive tool for the visual exploration of genomic data developed by the BROAD Institute [51, 52].

## Generation of the TFEB-3xFLAG dataset

HeLa cells were grown in Dulbecco’s Modified Eagle’s Medium (DMEM, Euroclone), supplemented with 10% heat- inactivated Fetal Bovine Serum (FBS, Hyclone). Cells were seeded in six-well plates at 10% confluence before transfection by using PolyFect Transfection Reagent (Qiagen) or Interferin (PolyPlus transfection) according to the manufacturer’s protocols. Transfectants for full-length TFEB-3xFLAG were selected with 1 mg/ml G418 (Sigma) to generate the TFEB-3xFLAG stable clones. To perform the transcriptome study, HeLa TFEB-FLAG stable clones [4] were grown in DMEM+ 10% FBS+ 1% Pen/Strep+ 1% Glutamine. The cells were seeded in triplicate in basal condition and RNA extraction was performed by using Qiagen Mini Kit, following the manufacturer’s instructions. 3 µg of total RNA was reverse transcribed to single-stranded cDNA with a special oligo (dT)24 primer containing a T7 RNA promoter site, added 3’ to the poly-T tract, prior to second strand synthesis (One Cycle cDNA Synthesis Kit by Affymetrix). Biotinylated cRNAs were then generated, using the GeneChip IVT Labeling Kit (Affymetrix). 20 µg of biotinylated cRNA was fragmented and 10 µg hybridized to the Affymetrix Human Genome U133 Plus 2.0 Array [HG-U133_Plus_2] as described in [53]. Low-level analysis to convert probe level data to gene level expression data was done using robust multiarray average (RMA) implemented using the RMA function of the Affymetrix package of the Bioconductor project [54] in the R programming language [55]. The low-level analysis for the BAMarray tool was performed using the MAS5 method, implemented using the corresponding function of the same Bioconductor package. For each gene, a *t*-test was used on RMA normalized data to determine if there was a significant difference in expression between the two groups of microarrays (TFEB-FLAG stable clones versus control cells). *P*-value adjustment for multiple comparisons was done with the FDR of Benjamini-Hochberg [56]. The BAM analysis was performed with BAMarray v3.0. The analysis was performed on MAS5 normalized array data using the default settings except for the following parameters: accuracy was set to high; clustering was set to manual with a value of 25, and variance was set to unequal. In this statistical analysis the threshold for statistical significance chosen was a FDR < 0.05. The Expression data from HeLa cells stable clones overexpressing TFEB-3xFLAG are available in the GEO database as GSE73054.

## Supplementary Material

Supplemental Material
